# Cultivation of the gut bacterium *Prevotella copri* DSM 18205^T^ using glucose and xylose as carbon sources

**DOI:** 10.1002/mbo3.1213

**Published:** 2021-06-27

**Authors:** Fang Huang, Roya R. R. Sardari, Andrius Jasilionis, Olof Böök, Rickard Öste, Ana Rascón, Lovisa Heyman‐Lindén, Olle Holst, Eva Nordberg Karlsson

**Affiliations:** ^1^ Division of Biotechnology Department of Chemistry Lund University Lund Sweden; ^2^ Aventure AB Lund Sweden; ^3^ Department of Food Technology, Engineering and Nutrition Lund University Lund Sweden

**Keywords:** anaerobe, bioreactor, cultivation, fermentation, *Prevotella copri*, type strain

## Abstract

*Prevotella copri* DSM18205^T^ is a human gut bacterium, suggested as a next‐generation probiotic. To utilize it as such, it is, however, necessary to grow the species in a reproducible manner. *Prevotella* *copri* has previously been reported to be highly sensitive to oxygen, and hence difficult to isolate and cultivate. This study presents successful batch cultivation strategies for viable strain inoculations and growth in both serum bottles and a stirred tank bioreactor (STR), without the use of an anaerobic chamber, as long as the cells were kept in the exponential growth phase. A low headspace volume in the STR was important to reach high cell density. *P*. *copri* utilized xylose cultivated in Peptone Yeast Xylose medium (PYX medium), resulting in a comparable growth rate and metabolite production as in Peptone Yeast Glucose medium (PYG medium) in batch cultivations at pH 7.2.Up to 5 g/L of the carbon source was consumed, leading to the production of succinic acid, acetic acid, and formic acid, and cell densities (OD_620 nm_) in the range 6−7.5. The highest yield of produced succinic acid was 0.63 ± 0.05 g/g glucose in PYG medium cultivations and 0.88 ± 0.06 g/g xylose in PYX medium cultivations.

## Graphical abstract

Successful batch cultivation strategies for reproducible growth of the anaerobe *Prevotella copri* DSM18205^T^, in both serum bottles and stirred tank reactor, are presented. Critical inoculum conditions were revealed and confirmed viable strain transfer without the use of an anaerobic chamber. The study provides fundamental knowledge to enable future commercial production.

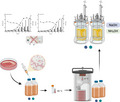

## 1 INTRODUCTION

It is well‐known that the human gut harbors millions of bacteria and their metabolic potential is immense. Dietary fiber in the human diet is the main source of accessible carbohydrate substrates for the gut microbiota, hence, using dietary manipulation of gut microbiota to increase the relative number of probiotic bacteria could contribute to the well‐being of the host. Probiotic bacteria protect humans from pathogens through a process known as colonization resistance (Sorbara & Pamer, 2019). Colonization resistance (van der Waaij et al., 1971) refers to the mechanism used by microbiota that is already present in the gut to maintain their presence, thereby avoiding colonization of the same intestinal sites by pathogens. In the absence of antibiotics, or under healthy conditions, the microbiota can effectively inhibit colonization and overgrowth of invading microbes associated with inflammation via specific interactions between the mucosal immune system and the microbiota. Different studies have shown that *Prevotella* is a common microbial genus in individuals with a plant‐based diet (high in fiber, and low in fats and protein), while an increase in *Bacteroides* is common in individuals with a Western diet (low in fiber, and high in fats and protein) (Arumugam et al., 2011; Costea et al., 2018; de Filippis et al., 2016, 2019; de Filippo et al., 2010; Rinninella et al., 2019; Smits et al., 2017). Growth of *Prevotella* spp. has also been found to be promoted by barley supplementation in healthy individuals and co‐occurred with improved glucose metabolism (Fehlner‐Peach et al., 2019; Kovatcheva‐Datchary et al., 2015; Sandberg et al., 2019). This finding indicates that the genus *Prevotella* plays a role in the dietary‐induced improvement in glucose metabolism in observed individuals (Kovatcheva‐Datchary et al., 2015; Pedersen et al., 2016; de Vadder et al., 2016) and it can be a potential probiotic, addressing glucose management. A recent human intervention study investigating barley as a prebiotic has also shown that a higher *Prevotella*/*Bacteroides* ratio may be beneficial to cardiometabolic regulation (Kovatcheva‐Datchary et al., 2015). However, the roles of bacteria classified under the *Prevotella* genus and their effects on humans are not clear since some studies show beneficial effects of *Prevotella* spp., while other studies suggest *Prevotella* spp. to be responsible for autoimmune diseases, insulin resistance and diabetes, and gut inflammation (Chang et al., 2019; Pedersen et al., 2016; Pianta et al., 2017; Scher et al., 2013).

Recently, one species in the *Prevotella* genus—*Prevotella copri*—was identified in responding to dietary fiber intake (de Filippis et al., 2019; Fehlner‐Peach et al., 2019). *P*. *copri* is also the predominant species of *Prevotella* in the intestinal tract of humans and potential next‐generation probiotic (Franke & Deppenmeier, 2018; Iljazovic et al., 2020). Whereas the abundance of *Prevotella* spp. in the gut microbiota after certain dietary interventions have been described (Precup & Vodnar, 2019), since its first isolation from human feces in Japan (Hayashi et al., 2007), still there is the need of further knowledge on the cultivation techniques to be able to utilize *Prevotella copri* strain DSM 18205^T^ (Hayashi et al., 2007). Studies of growth and growth conditions have thus far resulted in limited cell mass production (Fehlner‐Peach et al., 2019; Tramontano et al., 2018). Hence, further studies of the requirements to increase cell mass production are of fundamental importance to enable commercial production of the strain, for future use in food (or feed) applications. Moreover, phenotypic characterization at the applied conditions is valuable to increase the understanding of this bacterium.

This study aims to characterize how the growth of *P*. *copri* DSM 18205^T^ is influenced by different cultivation conditions. Essential insights are given here concerning the growth and propagation of the microorganism, as well as its metabolite production pattern.

## 2 MATERIALS AND METHODS

### 2.1 Materials

Trypticase peptone, peptone, yeast extract, meat extract, Tween 80, and Gram staining kits were supplied by Sigma‐Aldrich (St. Louis, MO, USA). Monosugar standards including glucose and xylose, a succinic acid standard, and short‐chain fatty acid standards including acetic acid, formic acid, lactic acid, propionic acid, butyric acid, and valeric acid were also supplied by Sigma‐Aldrich (St. Louis, MO, USA). Defibrinated sheep blood and Schaedler anaerobe agar were purchased from Oxoid (Basingstoke, UK). Anaerocult A was supplied by Merck (Darmstadt, Germany). Quick‐DNA Fungal/Bacterial Microprep kit was supplied by Zymo Research (Irvine, CA, USA) and QIAquick gel extraction kit from QIAGEN (Hilden, Germany). Cysteine hydrochloride and reagents for molecular biology including DreamTaq PCR Master Mix (2X) and nuclease‐free water were purchased from Thermo Fisher Scientific (Waltham, MA, USA). All chemicals were of analytical grade or higher quality.

### 2.2 Microorganism

*Prevotella copri* (CB7^T^ (Hayashi et al., 2007)) DSM 18205^T^ was purchased from the German Collection of Microorganisms and Cell cultures (DSMZ) (Braunschweig, Germany), and the bacteria were delivered on pre‐reduced Columbia blood agar plates. All inoculation procedures of *P*. *copri* took place using a class II laminar flow hood (NuAire, Plymouth, MN, USA) to prevent potential cross‐contamination. The cells were revived on defibrinated sheep blood, dehydrated Schaedler anaerobe agar plates. After inoculation from the initial plate aseptically (without any oxygen prevention), the inoculated plates were placed in a 2.5 L anaerobic jar using Anaerocult A to generate an anaerobic environment and incubated at 37℃. Bacterial colonies were visible after 12−48 hr of incubation.

### 2.3 Cultivation methods

#### 2.3.1 Medium preparation

A Peptone Yeast medium base without added sugar (PY medium) was initially prepared. Most of the constituents of the medium were dissolved in distilled water and boiled in the microwave until the color of resazurin (a selected oxygen indicator) was changed from pink to colorless. After boiling, the medium was cooled on wet ice while a stream of oxygen‐free CO_2_ was introduced. After that, L‐cysteine (0.5 g/L, as reducing agent), vitamin K_1_ (0.1% (v/v)), and hemin (with a final concentration of 5 mg/L) were added. Subsequently, the pH was adjusted to 7.2 with 8 N NaOH at room temperature, then the medium was distributed into serum bottles at room temperature and each serum bottle was flushed extensively with N_2_ for 3 min. The serum bottles were then sealed with a rubber bung and secured with metal caps and sterilized by autoclavation at 121℃ for 20 min. Glucose and xylose stock solutions for supplementation of PY medium were prepared separately with different concentrations ranging from 100 to 400 g/L, and filter sterilized into sterile serum bottles, flushed with filter‐sterilized oxygen‐free N_2_ for 10 min, and sealed. PY medium with added glucose is referred to as Peptone Yeast Glucose medium (PYG medium), and with added xylose as Peptone Yeast Xylose medium (PYX medium). PYG medium preparation followed DSMZ recommendations with minor modifications.

Horse serum was prepared by transferring the pre‐sterilized serum from its delivery bottle to a sterile serum bottle, where the same flushing step was applied, and the aliquoted serum was then kept at 4℃ until needed.

#### 2.3.2 Preparation of inoculum stock cultures

Before inoculation, the selected monosaccharide (either glucose or xylose with a final concentration of 5 g/L) and 5% (v/v) horse serum were added to serum bottles, resulting in 100 ml of the liquid medium in total. The inoculating syringes and needles were first aspirated with the nitrogen‐flushed PY medium (see above) to remove as much oxygen as possible from the inoculating materials. Thereafter, 5 mL of PY medium was transferred, using the pre‐flushed syringe and needle, to an agar plate with freshly grown colonies. The agar plate was shaken gently to suspend the cells, which were subsequently transferred using the same syringe and injected into a serum bottle supplemented with the selected monosaccharide and horse serum. The serum bottles were incubated at 37℃ without agitation for at least 12 hr. Cell growth was monitored by measuring the optical density at 620 nm (OD_620 nm_), and in the exponential phase (approximate OD_620 nm_ 4−5), 1 ml of the culture was added to 0.5 ml of 50% (v/v) pre‐sterilized glycerol (final concentration of 15% (v/v)) using a 2 ml cryotube, gently mixed and kept as a stock culture at −80℃ without any oxygen prevention. The rest of the cells were used to inoculate a series of cultures.

#### 2.3.3 Culture purity assessment

**FIGURE A1:**
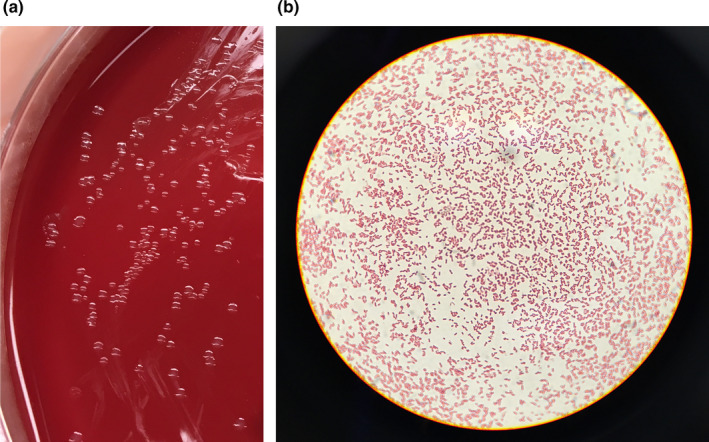
(a) *Prevotella copri* DSM 18205^T^ grown on Columbia blood agar plate. (b) Gram staining and microscope observation (100× magnification) of *Prevotella copri* DSM 18205^T^

The culture purity of *P*. *copri* DSM 18205^T^ was routinely assessed by evaluating phenotypic features of the colonies, complemented by sequence analysis of the 16S rRNA gene (described below). All culture handling and culture sampling was made aseptically. Schaedler agar plates, cultivated anaerobically, were used to assess culture purity by determining and comparing colony phenotype characteristics. Microscopy was also used to visually evaluate culture purity after anaerobic cultivation on Schaedler agar as well as in PYG medium. Gram staining was routinely performed (Figure A1) using a Gram staining kit.

**FIGURE A2:**
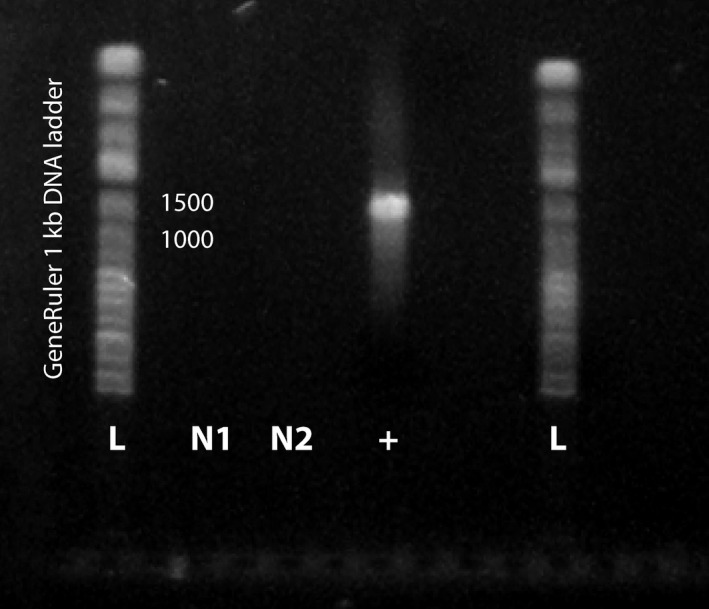
Agarose gel electrophoresis of 16S rRNA genes amplified from the bacterial cultures. PCR amplified products were run on 1% (w/v) agarose gel in TAE buffer staining gel with GelRed. Lane (L) GeneRuler 1 kb DNA Ladder (Thermo Fischer Scientific). Lane (N1) the negative control consisted of the PCR without template DNA; Lane (N2) the negative control consisted of template DNA without primers. Lane (+) PCR amplified 16S rRNA gene of the respective bacterial isolates

**FIGURE A3:**
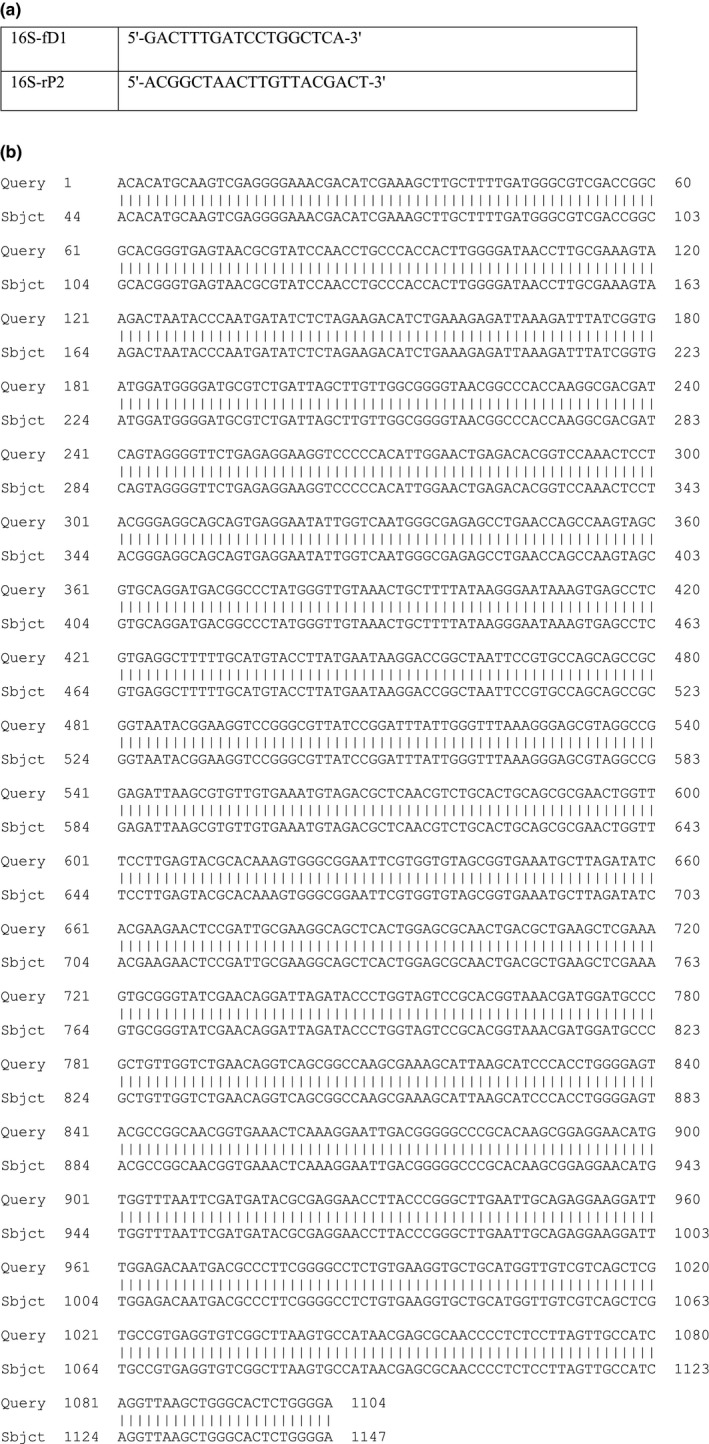
16S rRNA primers (a) and alignment of the best match of the Blast nucleotide search in GenBank (b) using the PCR amplified 16S rRNA sequence as query. The best match (sbjct) was the 16S rRNA of *Prevotella copri* DSM18205^T^ (GenBank accession code NR_113411.1)

A Quick‐DNA Fungal/Bacterial Microprep kit was used for total DNA isolation from culture samples. DreamTaq PCR Master Mix (2X) was used for 16S rRNA gene amplification using the universal primer pair fD1 and rP2 (Weisburg et al., 1991). The volume of each PCR reaction was 50 μl: 0.25 mmol of each primer and 10 ng of template in nuclease‐free water. The PCR was conducted as follows: 95℃ initial denaturation, 3 min; 30 cycles of 95℃ denaturation, 30 s; 60℃ annealing, 30 s; and 72℃ extension, 2 min, with a final extension at 72℃ for 10 min. The PCR products were fractionated by 1% (w/v) agarose gel (Figure A2) electrophoresis in a TAE buffer system applying 10 V/cm. Products of the expected length were excised from the gel using a QIAquick gel extraction kit and sequenced by Eurofins Genomics Sweden (Solna, Sweden). The obtained sequences (Figure A3) were aligned with the *P*. *copri* DSM 18205^T^ 16S rRNA gene (NCBI Ref Seq: NR_040877.1).

#### 2.3.4 Preparation of inoculum at defined growth phases and volumes

Cells were collected from the colonies on agar plates and transferred using syringes and needles (pre‐aspirated/flushed as described above) into serum bottles, to be used in the inoculation of a series of cultures. The inocula were collected at different time intervals: (a) when the OD_620 nm_ reached 4−5 after ~5 hr, (b) when the maximum OD_620 nm_ was reached (between 6−7) after 24 hr, and (c) after 48 hr. The cultures were inoculated up to 10% (v/v) with the inoculum a, b, and c, separately, and the growth of cells in each bottle was monitored by measuring OD_620 nm_ after 24 hr.

The inoculum culture (100 ml) in PYG medium (supplemented with 5 g/L glucose and 5% (v/v) horse serum) was prepared anaerobically (as described above) in a 150 mL serum bottle. Once the OD_620 nm_ of culture reached 4−5, the cells were inoculated into new 40 mL serum bottles containing 20 mL of PYG medium using the following inoculum culture volumes: 1% (v/v), 2% (v/v), 4% (v/v), 6% (v/v), 8% (v/v), and 10% (v/v), separately. The inoculated cultures were incubated at 37℃ without agitation. Samples were taken every hour monitoring OD_620 nm_ of each cultivation. The experiments were carried out in duplicate.

#### 2.3.5 Sampling techniques

An inoculum (100 ml) in PYG medium (supplemented with 5 g/L glucose and 5% (v/v) horse serum) was prepared anaerobically (as described above) in a 150 ml serum bottle. Once the OD_620 nm_ reached 4−5, the cells were inoculated up to 8% (v/v) into 13 new 40 ml serum bottles containing 20 ml of PYG medium and three new 100 ml serum bottles containing 100 ml of PYG medium. The inoculated serum bottles were incubated at 37℃ without agitation. Samples for OD_620 nm_ measurements were taken according to two different schemes: (a) between batch sampling that was based on the usage of separate 40 ml serum bottles, harvested every hour for the OD_620 nm_ measurement, or (b) within batch sampling, based on repeated sampling in single cultivation (100 ml serum bottle), in which sampling was made by the withdrawal of 1 ml culture every hour for the OD_620 nm_ measurement. The syringes and needles were aspirated/flushed with PY medium as blank medium twice before injection of inoculum and prior to culture sampling.

#### 2.3.6 Growth of *P. copri* DSM 18205^T^ at different pH values

Growth experiments at different pH were carried out in duplicate in serum bottles, using a PYG medium containing 5 g/L glucose (as described above), in which the initial pH was adjusted to 5.5, 6.0, 6.5, and 7.2. Samples were taken every hour monitoring OD_620 nm_ of each cultivation.

#### 2.3.7 Growth on glucose and xylose in serum bottles

Two different inocula (100 ml) in PYG medium (supplemented with 5 g/L glucose and 5% (v/v) horse serum) or PYX medium (supplemented with 5 g/L xylose and 5% (v/v) horse serum), were separately prepared (as described above) in two 150 ml serum bottles. Once the OD_620 nm_ of the preculture reached 4−5, 4% (v/v) portions of the cells were inoculated (using pre‐aspirated syringes and needles) into new 150 mL serum bottles containing 100 ml of PY medium (control), PYG medium, or PYX medium, supplemented with 5 g/L, 10 g/L, and 20 g/L of glucose or xylose, respectively. The serum bottles were incubated at 37℃ without agitation. Samples were taken at the end of each cultivation for OD_620 nm_ measurement.

During our work, several types of controls have been used, including medium blanks (without cells) or cultivation trials without added carbon sources (negative controls).

#### 2.3.8 Cultivation of *P. copri* DSM 18205^T^ in bioreactor

Batch cultivation of *P*. *copri* DSM 18205^T^ was performed in a Multifors 2 stirred tank bioreactor (Infors HT, Bottmingen, Switzerland), with a total reactor volume of 1.4 L. Prepared PYG medium (as described above) was transferred to the bioreactor, which was sealed and sterilized by autoclavation. After sterilization, the bioreactor was placed in a laminar flow hood, and the inlet and outlet sterile filters were connected to the bioreactor tubings. N_2_ gas (100 kPa) was then bubbled through the medium in the bioreactor while equilibrating to 37℃. After temperature equilibration, 5 g/L glucose and 5% (v/v) horse serum were added using a pre‐aspirated sterile syringe and needle. The pH was maintained at 7.2 by adding base (see below). The inoculum was prepared in a serum bottle from the stock culture and incubated to reach the exponential phase (OD_620 nm_ 4−5), at which the inoculum up to 4% (v/v) was added to the bioreactor using a pre‐aspirated syringe and needle. To avoid oxygen in the bioreactor, filter‐sterilized N_2_ was constantly introduced in the headspace of the reactor. The temperature of the bioreactor was set to 37℃ and the agitation speed was set to 80 rpm. A series of cultivations were performed using this set‐up, with two parameters varied: (a) the base for pH control, which was either 1 M NaOH, or 4 M NH_4_OH, and (b) the headspace volume, by setting the working volume to either 0.5 or 1 L.

### 2.4 Analytical methods

#### 2.4.1 Determination of cell density and cell dry weight

The cell suspension OD_620 nm_ was measured, after adequate dilution with distilled water, using a WPA Biowave II UV/Visible spectrophotometer (Biochrom, Cambridge, UK).

Cell dry weight was determined by analytical weighing. Two milliliters of the culture liquid containing *P*. *copri* cells was centrifuged at 17,135×*g* for 5 min. Obtained cell pellets were separated from the supernatant and washed twice with distilled water, centrifuged repeatedly discarding water. Cell pellets were then resuspended in 0.5 ml of distilled water and the suspension was transferred to a pre‐weighed aluminum boat that had been dried overnight in an oven at 100℃. The boat loaded with suspension was then placed in an oven at 100℃ and dried overnight. During the drying time, the boats containing cells were measured periodically until no further decrease in dry weight was observed. The cell dry weight was estimated by calculating the difference between the aluminum boat weight before and after adding the cell suspension.

#### 2.4.2 Determination of glucose and xylose concentration in the culture medium

Glucose and xylose were determined using a high‐performance anion‐exchange chromatography (HPAEC) Dionex system operated by Chromeleon 7.0, (Thermo Fisher Scientific, Waltham, MA, USA) with a Dionex CarboPac PA20 IC analytical column (Thermo Fisher Scientific, Waltham, MA, USA), coupled to a Dionex CarboPac PA20 IC guard column (Thermo Fisher Scientific, Waltham, MA, USA). Three pumps were used for three different eluents: pump A (ultrapure water), pump B (2 mmol NaOH), and pump C (200 mmol NaOH). Separation occurred during 23 min running time using a mixture of A (62.5%) and B (37.5%) with an isocratic flow of 0.5 ml/min and thereafter the column was regenerated with C (100%) for 2 min at the same flow rate. The analytes were detected with a Dionex ED40 electrochemical detector (Thermo Fisher Scientific, Waltham, MA, USA). Before injection, the cell‐free supernatant was filtered through a 0.2 μm polypropylene filter after adequate dilution. Glucose or xylose content in each sample was analyzed and compared to the corresponding external monosugar standards.

#### 2.4.3 Analysis of organic acids

Analysis of organic acids was carried out by high‐performance liquid chromatography (HPLC). After adequate dilution, 1 ml of a cell‐free sample was acidified with 20 μl of 20% (v/v) H_2_SO_4_, and filtered through a 0.2 μm polypropylene filter, before analysis in a Dionex UltiMate 3000 RSLC HPLC system (Thermo Fisher Scientific, Waltham, MA, USA) connected to a Shodex RI‐101 differential refractive index detector (Showa Denko, Tokyo, Japan). The organic acids were separated using the Aminex HPX‐87H analytical column coupled to a guard column (Bio‐Rad, Hercules, CA, USA). A series of standard solutions including formic acid, acetic acid, lactic acid, butyric acid, succinic acid, propionic acid, and valeric acid were prepared and analyzed together with the samples. The temperature was set to 40℃ and the mobile phase consisted of 5 mmol H_2_SO_4_ with a flow rate of 0.5 ml/min. A small amount of acetic and succinic acid was present in the PY medium (without cells), and was subtracted when organic acids were calculated.

#### 2.4.4 Yield calculation

The yield (Y) of secondary metabolites (m) produced per unit of substrates (S) consumed was calculated. In this study, the substrate was either glucose or xylose. The amount of substrate consumed is the difference between the initial concentration (S_0_) and the concentration of substrate left after the growth period (S), using the formula: Y = (m‐m_0_)/(S_0_‐S) (Lele & Gwatve, 2014).

## 3 RESULTS

### 3.1 Inoculum preparation, inoculation, sampling, and storage

A reproducible inoculation strategy of *P*. *copri* DSM 18205^T^ cells was first developed for growth on blood agar plates and culture transfer to serum bottles containing PYG medium. The procedure did not demand the use of an anaerobic chamber, but to assure good cell viability, the disposable syringes and needles were pre‐flushed with a nitrogen‐flushed PY medium. After inoculation, plates with freshly grown colonies could be stored in an anaerobic jar at 4℃ for 1 to 2 weeks without loss of viability, and during this period were repeatedly used to inoculate fresh blood agar plates.

The purity of the resulting *P*. *copri* cultures was routinely confirmed by both visual inspection of the colonies and cells, and PCR amplification of 16S rRNA gene from the total DNA isolated from the cultures followed by sequencing. The *P*. *copri* DSM 18205^T^ colonies were round, low convex, translucent, with a smooth surface (Hayashi et al., 2007). Gamma‐type hemolytic reaction (no hemolysis) on the surface of blood agar was also observed. Aging or large colonies grown on agar plates could result in an opaque appearance, and the colonies appeared brown‐gray after incubation for 48 hr under anaerobic conditions.

**FIGURE A4:**
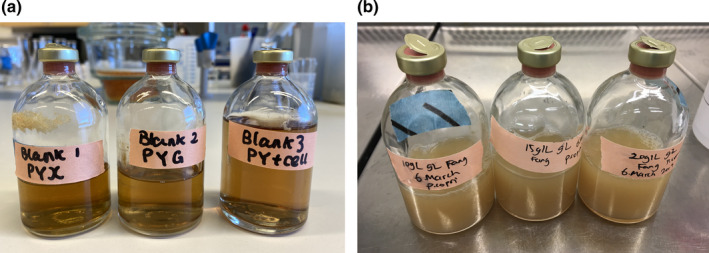
(a) Negative controls, cultivations without either carbon source or cells (b) *Prevotella copri* DSM 18205^T^ grown in liquid medium

The cells were initially only reproducibly proliferated after inoculation from blood agar plates (plate to plate (P‐P) or plate to liquid cultures (P‐L), using serum bottles containing liquid PYG medium). Inoculation from liquid PYG medium was more troublesome (liquid to liquid (L‐L), or liquid to plate (L‐P)). This was found to be growth phase‐related, not surprisingly, showing that the growth phase of cells in liquid cultures was more uniform than in plate cultures. To shed more light on the importance of the growth phase for successful inoculation, a set of cultivations in the liquid medium was made, where the cells were harvested in the exponential phase (OD_620 nm_ 4−5) and stationary phase (OD_620 nm_ 6−7, after 24 and 48 hr), and used as inocula in subsequent cultivations. Growth within 24 hr after L‐L (Figure A4) or L‐P (Figure A1) culture transfer was only obtained from inoculum cells harvested in the exponential phase (OD_620 nm_ 4−5). In contrast, inoculum cultures that had reached the stationary phase (OD_620 nm_ 6−7) generally failed to proliferate (especially if the cells were harvested after 48 hr).

**FIGURE 1:**
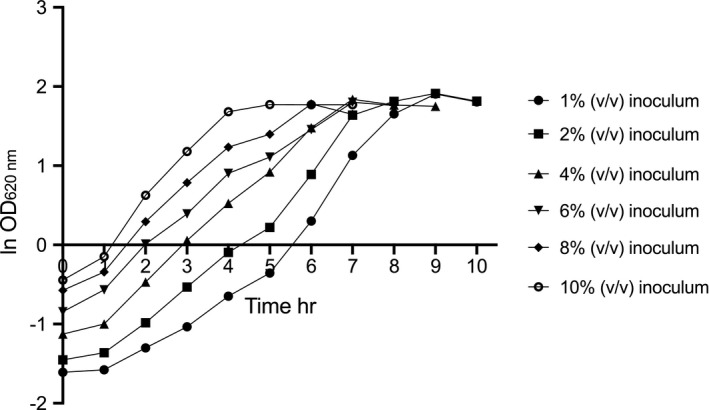
Growth profile of *Prevotella copri* DSM 18205^T^ by anaerobic cultivation in Peptone Yeast Glucose medium. Symbols indicate (●) 1% (v/v) inoculum; (■) 2% (v/v) inoculum; (▲) 4% (v/v) inoculum; (▼) 6% inoculum; (♦) 8% (v/v) inoculum; (○) 10% (v/v) inoculum

The influence of the inoculum volume (using exponential phase culture harvested at OD_620 nm_ 4−5) on growth in liquid PYG medium in serum bottles was investigated. The inoculation culture volume was varied from 1 to 10% (v/v) (Figure 1), but was not shown to be of major importance. There was no obvious lag phase in the cultures, but the specific growth rate was initially lower when a smaller inoculum volume was used. In addition, the final cell density was in the same range in all cases, with a shorter growth phase when a high inoculum volume of up to 10% (v/v) was used, favoring intermediate inoculum volumes (Figure 1).

The highest specific growth rate of 0.52 h^−1^ during a 5 h exponential phase was monitored after using a 4% (v/v) inoculum because it ensured a quick onset of the exponential stage with a stable growth rate; 4% (v/v) inoculum size was rendered a suitable volume for all further cultivations.

**FIGURE 2:**
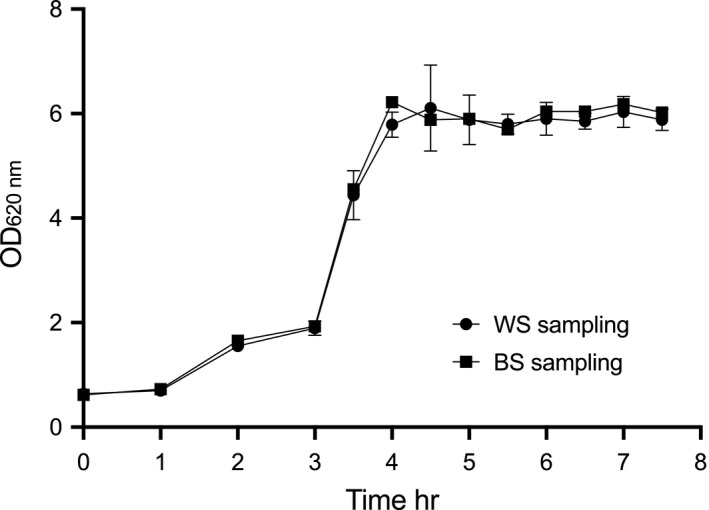
Comparison between batch sampling (BS), using 13 flasks, and within batch sampling (WS) using one flask, cultivated anaerobically. Symbols indicate: (●) *Prevotella copri* DSM 18205^T^ growth represented as OD_620 nm_ measured by (WS) sampling and (■) *P*. *copri* DSM 18205^T^ growth represented as OD_620 nm_ measured by (BS) sampling

The effect of using within or between batch sampling schemes was taken into consideration for correct quantification of cultivation parameters in cultures grown at anaerobic conditions. As sampling must be performed while preventing the introduction of oxygen to the culture medium, two parallel experiments were carried out to evaluate the effect of either between batch sampling (BS) using a single sampling occasion from many parallel bottles and within batch sampling (WS) using repeated sampling from one bottle. The inoculum OD_620 nm_ was 4.6 in both cases. The results demonstrated that there was no difference between the two sampling schemes. *P*. *copri* DSM 18205^T^ reached the stationary phase after 4 h of cultivation with a maximum OD_620 nm_ of 6.2 in the WS cultivation and 6.1 in BS (Figure 2).

### 3.2 Serum bottle batch cultivations with glucose and xylose as carbon source

**FIGURE 3:**
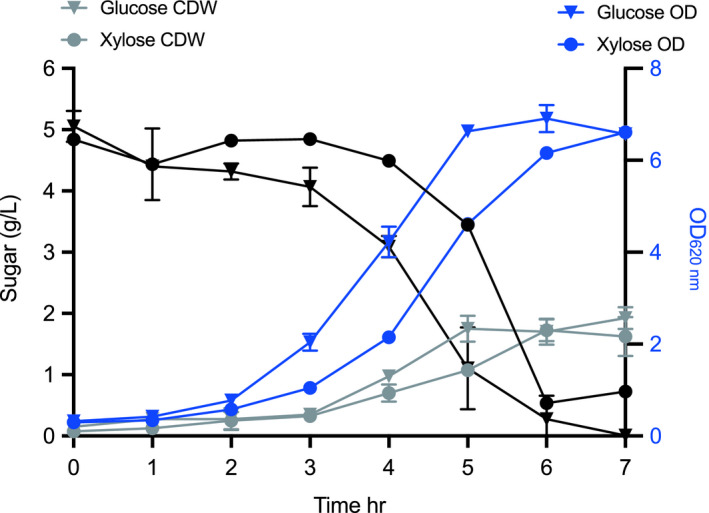
Growth profile of *Prevotella copri* DSM 18205^T^ in serum bottles in Peptone Yeast Glucose medium and Peptone Yeast Xylose medium. Symbols indicate: (▼) *P*. *copri* DSM 18205^T^ growth represented as OD_620 nm_ in PYG medium; (●) *P*. *copri* DSM 18205^T^ growth represented as OD_620 nm_ in PYX medium; (▼) *P*. *copri* DSM 18205^T^ cell dry weight in PYG; (●) *P*. *copri* DSM 18205^T^ growth represented as cell dry weight in PYX; (▼) glucose consumption; (●) xylose consumption medium

**FIGURE 4:**
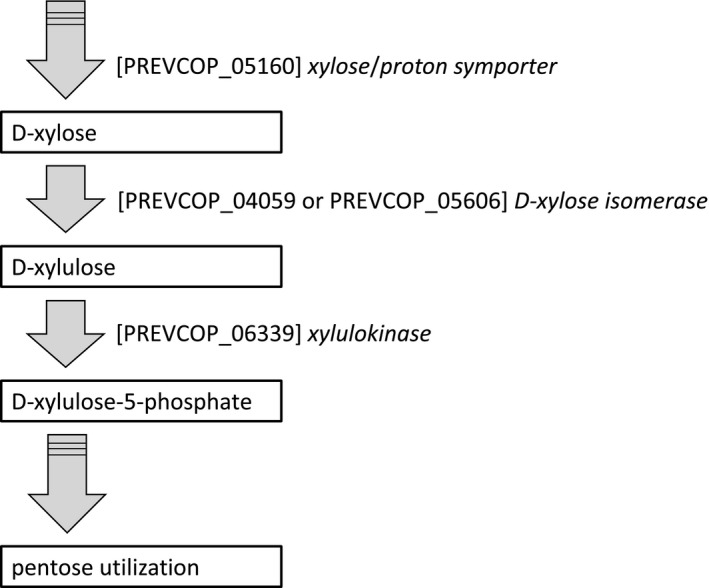
Suggested D‐xylose utilization pathway in *Prevotella copri* DSM 18502^T^. Enzyme designations correspond to strains genome annotation. Isomerases PREVCOP_04059 and PREVCOP_05606 are isomerases homologs that may catalyze the first step in the pathway as typical D‐xylose isomerase is absent in *P*. *copri* DSM 18502^T^ genome. Adapted from (Garschagen et al., 2021) with additions from (Takahashi & Yamada, 2000)

Independent batch cultivations were first carried out in serum bottles at 5 g/L using either xylose or glucose as the carbon source in the PY medium (Figure 3). Similar final cell densities were observed for both carbon sources (OD_620 nm_ 6.57 ± 0.29 in PYG medium corresponding to 1.9 g/L cell dry weight, and OD_620 nm_ 6.65 ± 0.09 in PYX medium corresponding to 2.1 g/L cell dry weight), with little difference in specific growth rate (0.59 h^−1^ in PYG medium, compared to 0.56 h^−1^ in PYX medium), showing similar growth behavior on both monosaccharides. This confirms xylose utilization, which has been proposed to follow the pentose phosphate pathway (Garschagen et al., 2021; Gupta et al., 2015). To date, however, xylose isomerase is not unambiguously identified in *P*. *copri*, although two alternative potential isomerase homologs are available in the genome of strain DSM 18205^T^ (Figure 4). In our work, complete substrate utilization was seen in PYG medium, while xylose consumption stopped at the onset of the stationary phase, leaving 0.7 ± 0.1 g/L of unconsumed xylose (Figure 3).

Further increase in the initial glucose concentration (10 or 20 g/L) did not result in growth inhibition but led to incomplete substrate utilization and a limited increase in the final OD_620 nm_. The final OD_620 nm_ was 7.1 and 7.2, after growth in 10 and 20 g/L glucose‐supplemented cultures, leaving an unconsumed total of 6.3 and 15.8 g/L glucose, respectively, showing that the medium is not balanced for carbohydrate additions above 5 g/L.

**FIGURE 5:**
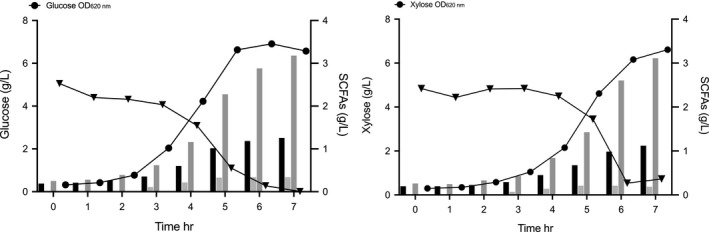
Organic acid profile and sugar consumption in anaerobic cultivation of *Prevotella copri* DSM 18205^T^. Symbols indicate (●) *P*. *copri* DSM 18205^T^ growth represented as OD_620 nm_; (▼) sugar consumption, black column represented acetic acid, light gray column represented formic acid, and dark gray represented succinic acid

For further investigation of growth characteristics of *P*. *copri* DSM 18205^T^, the bacterial metabolism end products from cultivations in PYG medium and PYX medium were determined. Succinic acid, acetic acid, and formic acid were found to be produced, both when glucose or xylose was used as substrates for cultivation. Significant production of acetic acid and succinic acid was observed after only 2 hr of cultivation, and the organic acids increased gradually in both glucose and xylose‐supplemented cultures (Figure 5). A continued increase in the stationary phase samples was also observed, leading to the production of succinic acid with yields of 0.63 ± 0.05 g/g and 0.88 ± 0.06 g/g in PYG medium and PYX medium, respectively, followed by acetic acid with yields of 0.25 g/g and 0.30 ± 0.01 g/g in PYG medium and PYX medium, and formic acid with yields of 0.07 ± 0.02 g/g and 0.05 g/g in PYG medium and PYX medium, respectively. No production of propionic acid or butyric acid was detected in the *P*. *copri* cultures. No bacterial growth was observed in the blank controls or the negative controls.

### 3.3 **The influence of pH on** *P. copri* **DSM 18205^T^ growth and metabolite production**

The effect of pH on bacterial growth was screened to analyze the need for pH adjustments in simple batch cultivations in the serum bottles (Table 1). The result showed that growth, substrate consumption, and metabolite production decreased with decreasing pH, in the pH range 7.2−5.5.

**TABLE 1:** Evaluation of the influence of pH on growth and metabolite production of *Prevotella copri* DSM 18205^T^ cultivated in serum bottles

	pH 5.5	pH 6	pH 6.5	pH 7.2
Maximum OD_620 nm_	1.38 ± 0.33	3.50 ± 0.40	3.62 ± 0.23	6.91 ± 0.30
Consumed glucose (g/L)	0.79 ± 0.01	2.97 ± 0.01	2.35 ± 0.55	5.04 ± 0.25
Acetic acid (g/L)	0.32 ± 0.03	0.76 ± 0.01	0.82 ± 0.40	1.25 ± 0.06
Formic acid (g/L)	Not detected	0.08 ± 0.01	0.11 ± 0.01	0.34 ± 0.10
Succinic acid (g/L)	0.90 ± 0.13	2.11 ± 0.07	2.33 ± 0.59	3.18 ± 0.08

### 3.4 Cultivation of *P. copri* DSM 18205^T^ in bioreactor

A series of fermentations in PYG medium in stirred tank bioreactors (STR) were evaluated, using two types of alkaline compounds (1 M NaOH, and 4 M NH_4_OH) for pH control, at two working volumes (0.5 and 1 L), thus varying the headspace volume (0.9 and 0.4 L) to investigate the need to limit oxygen in the reactor. The two cultivations with working volumes of 0.5 L but with different bases for pH control had similar but low cell yields, with rather low final cell densities, and low consumption of glucose (Table 2), in contrast to the results obtained in serum bottles. The two bioreactor cultivations with working volumes of 1 L grew to higher cell densities, and consumed more of the glucose, showing the importance of limiting oxygen for growth (Table 2).

**TABLE 2:** Organic acid production and sugar consumption in bioreactor fermentation of glucose by *Prevotella copri* DSM 18205^T^ at working volumes of 0.5 and 1 L. 5 g/L of glucose and 5% (v/v) horse serum were supplied to all experiments, prepared base solutions for controlling pH at 7.0 were 1 M NaOH or 4 M NH_4_OH

Working volume (L)	pH	Base	Maximum OD_620 nm_	Consumed sugar	Acetic acid	Formic acid	Succinic acid
0.5	7	NaOH	3.3	1.3	0.47	0.33	1.95
		NH_4_OH	3.3	1.1	1.58	0.21	0.73
1	7	NaOH	7.12	(6.5–2.0) = 4.5	0.28	0.12	0.57
		NH_4_OH	7.44	(6.5–2.7) = 3.8	0.34	0.14	0.65

**FIGURE 6:**
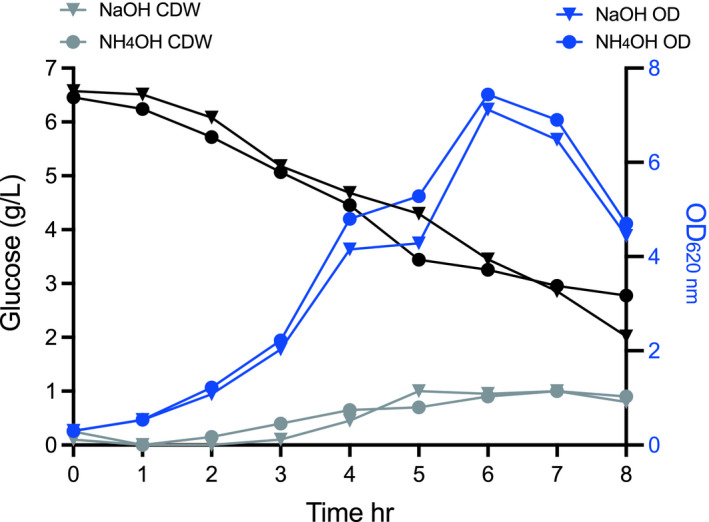
Growth profile of *Prevotella copri* DSM 18205^T^ in a bioreactor with the working volume of 1 L. Two independent fermentations were carried out using two different bases: 1 M NaOH or 4 M NH_4_OH. Symbols indicate (▼) *P*. *copri* DSM 18205^T^ growth represented as OD_620 nm_ in NaOH controlled PYG medium; (●) *P*. *copri* DSM 18205^T^ growth represented as OD_620 nm_ in NH_4_OH controlled PYG medium; (▼) *P*. *copri* DSM 18205^T^ growth represented as cell dry weight in NaOH controlled PYG medium; (●) *P*. *copri* DSM 18205^T^ growth represented as cell dry weight in NH_4_OH controlled PYG medium; (▼) glucose consumption in NaOH controlled PYG medium; (●) glucose consumption in NH_4_OH controlled PYG medium

The choice of the base did not lead to major differences in 1 L cultivations. The culture reached a maximum OD_620 nm_ 7.1 (with a 1 g/L cell dry weight) using 1 M NaOH as pH control, while the fermentation using 4 M NH_4_OH reached a maximum OD_620 nm_ 7.4 (again with 1 g/L cell dry weight), after 6−7 hr of cultivation (Figure 6).

**FIGURE 7:**
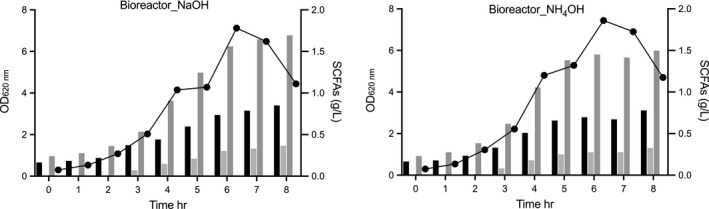
Organic acid production profile of *Prevotella copri* DSM 18205^T^. Symbols indicate (●) *P*. *copri* DSM 18205^T^ growth represented as OD_620 nm_. The black column represents acetic acid, the light gray column represents formic acid, and dark gray represents succinic acid

The sugar consumption analysis showed incomplete utilization of glucose with 2.0 g/L and 2.7 g/L of glucose remaining in cultivations with pH control using NaOH and NH_4_OH, respectively. As the sugar was not completely consumed in the bioreactor, even at 1 L working volume, a lower level of metabolites was foreseen and observed (Figure 7), but the amount produced per cell was in the same range as in previous production trials. Interestingly, the production ratio of organic acids was affected by the base used for pH‐control in the reactors run with low working volume (0.9 L headspace), resulting in lower succinic acid and higher acetic acid production (Table 2). In our study, *P*. *copri* DSM 18205^T^ growth in the serum bottles was without agitation, while in the bioreactor an agitation speed of 80 rpm was used to assure mixing of the cells and medium components, which, however, was not varied between cultivations in different working volumes.

## 4 DISCUSSION

The crucial step for reviving *P*. *copri* DSM 18205^T^ without the use of an anaerobic chamber especially during the L‐L or L‐P cultivation transfers was firstly revealed in this study. The results demonstrate the importance of maintaining cells in the exponential growth phase for viable *P*. *copri* DSM 18205^T^ cell culture transfers. The genus *Prevotella* is reported to include obligate anaerobic bacteria (Zhou & Li, 2015) that may be killed by exposure to air. However, we found that *P*. *copri* does seem to tolerate some degree of oxygen exposure because (a) there was oxygen present in the stock cultures, and (b) laboratory work routinely carried out aseptically in a laminar flow hood is applicable. Instead, the growth phase of the cells in the inoculum culture plays a significant role in maintaining the proliferation of cell cultures.

The duration of the lag phase and the shape of the exponential phase in the growth curve have been reported to depend on the physiological state of the cells in the inoculum, for example, the fraction of viable cells in the inoculum, and the handling of the inoculum cultures (Hornbaek et al., 2004; Leite et al., 2017; Zhang et al., 2002). In this study, only small differences were seen as a consequence of different inoculum sizes, which indicates uniform viability and growth phase in the inoculum. The lack of difference between the cultures subjected to BS (between batch) and WS (within batch) sampling further supports the critical point of harvesting the cell during the exponential growth phase for proliferation purposes.

Bacterial growth using the pentose xylose as the carbohydrate source has previously been reported for other species from the *Prevotella* genus (Hector et al., 2013). The independent batch cultivations in serum bottles and the bioreactor, reported here, using either xylose or glucose as a carbon source in PY medium, confirmed the utilization of xylose by *P*. *copri* DSM 18205^T^, and a similar growth behavior was shown on both monosaccharides.

Anaerobic catabolic pathways are known to result in the production of organic acids and alcohols in a number of microorganisms; and among the organic acids, short‐chain fatty acids (SCFAs) are abundant bacterial products derived from commensal bacterial fermentation of dietary fibers in intestines (den Besten et al., 2013). The end products of *P*. *copri* cultivation experiments in PYG medium and PYX medium were succinic acid, acetic acid, and formic acid. In a recent study, transcriptome profiling indicated that the central metabolic pathways of *P*. *copri* are based on glycolysis and succinate production from fumarate (Franke & Deppenmeier, 2018). In addition, the degradation of pyruvate leads to the accumulation of acetate and formate. Our findings fit well with this product pattern. No production of propionic acid was detected in the *P*. *copri* cultures, in accordance with the observation by Franke and Deppenmeier (2018) that *P*. *copri* lacks the genes encoding the key enzymes methylmalonyl‐CoA mutase, methylmalonyl‐CoA epimerase, and succinate: methylmalonyl‐CoA transferase.

Increasing the initial glucose concentration in simple batch cultivation in serum bottles did not result in growth inhibition, which shows the potential to further increase the cell density by other modes of operation (e.g., fed‐batch or sequential batch) or medium balancing, considering nitrogen content, as well as vitamin K_1_ and hemin that both are reported to play important roles in the bacterial proliferation (Franke & Deppenmeier, 2018).

In this work, the cells grew to higher cell densities compared to previous works (Fehlner‐Peach et al., 2019; Tramontano et al., 2018). There may be three possible reasons for this: (a) the medium in this work has been balanced to include a higher amount of the carbohydrate source, (b) the oxygen level in the cultivation liquid was kept low, by nitrogen flushing prior to inoculation, and in stirred tank reactors it was of importance to keep the headspace low, (c) the growth phase of the inoculum cultures has been controlled, which was proven to be very important.

The pH is a crucial parameter for any fermentation process (Baldi et al., 2019; Park et al., 2016), and is affected by the organic acids produced by the bacteria. Growth constraints due to the excessive acidification in the system were shown here, which increased our interest in applying stirred tank bioreactor cultivations with pH control, employing a pH adjustment strategy through the addition of alkaline solutions. This strategy also allows the scale‐up of the cultivation, simplifying harvesting of higher cell mass and higher yield of any metabolic target compound, which is a crucial step to obtain significant amounts of selected microbes and their products (Fan et al., 2017; Ren et al., 2006; Ron et al., 2019). Despite all previous reports on the challenge of keeping an oxygen‐free environment to allow growth of *P*. *copri*, our growth trials in the STR system were successful, showing that the aerotolerance of *P*. *copri* DSM 18205^T^ was sufficient for STR cultivations.

The series of fermentations in PYG medium in stirred tank bioreactors, however, revealed that cultivations with a low working volume (0.5 L) and a larger headspace resulted in rather low final cell densities and low consumption of glucose, in contrast to the results obtained in serum bottles. The cell density and glucose consumption were significantly improved when the working volume was increased (1 L), which limited the headspace during the cultivation. This shows the importance of limiting oxygen for successful cell proliferation of *P*. *copri* DSM 18205^T^ in the system.

Further improvements may still be necessary for optimal yields, as the cell dry weight obtained per volume was lower than in the serum bottle cultivations. This indicates that the cells may be under metabolic stress at the conditions in the STR; for example cell lysis (visible as a reduction in OD, Figure 6, 7,) repeatedly occurred in the early stationary phase in the cultivation trials in the STR. The reason for this is not clear, and corresponding lysis was not observed in serum bottle cultivations. Moreover, the choice of base for the pH control (which did not lead to major differences in the metabolite profile in 1L cultivations), affected the production ratio of organic acids in the reactors that ran at low working volume (0.5 L).

In addition, the ratio between OD_620 nm_ and the dry weight changed compared to the cultivations in serum bottles, raising the possibility that some compound affecting the cell density measurements may be produced. The cell dry weight ratio of OD_620 nm_ in serum bottle cultivation is in line with those obtained in a previous study on *P*. *copri* DSM 18205^T^ (Franke & Deppenmeier, 2018). This leads us to hypothesize that the bacteria produce something that affects the OD/dry weight ratio in the STR, for example, some type of storage polysaccharide, which previously has been reported to be intracellularly located in other *Prevotella* spp (Takahashi et al., 2000). Many factors can affect bacterial growth and metabolite production (Zhou et al., 2018), including aeration, agitation speed, pH, and working volume (as seen here). Agitation speed could also have an impact on bacterial growth and production, as exemplified in cultivations of *Bacillus licheniformis* BT5.9, where maximal amylase production and maximal growth were achieved at an agitation speed not higher than 100 rpm (Ibrahim et al., 2013). In our study, *P*. *copri* DSM 18205^T^ growth in the serum bottles was without agitation, while the agitation speed of 80 rpm was used in the bioreactor, which, however, was not varied between cultivations in different working volumes.

## 5 CONCLUSION

The study revealed several important findings to allow the cultivation of the anaerobic bacterium *P*. *copri* DSM 18205^T^ in serum bottles as well as in a stirred tank bioreactor. The strain was found to be oxygen tolerant during inoculations and culture transfers, thus simplifying handling, but crucial for successful inoculations was the use of exponential phase culture to maintain viable cell cultures. The specific growth rate of *P*. *copri* was in the same range using either glucose or xylose as the carbon source, and in both cases yielded a significant production of succinic acid. In addition, the successful scaling up of the cultivation to a stirred tank bioreactor provides a solid base for studying the bacterium and opens up opportunities for more efficient production.

## 6 ETHICS STATEMENT

None required.

## CONFLICT OF INTEREST

None declared.

## AUTHOR CONTRIBUTION

**Fang Huang:** Data curation (lead); Formal analysis (lead); Investigation (lead); Methodology (equal); Validation (equal); Visualization (equal); Writing‐original draft (equal); Writing‐review & editing (equal). **Roya R. R. Sardari:** Conceptualization (equal); Data curation (supporting); Formal analysis (supporting); Investigation (equal); Methodology (equal); Supervision (equal); Validation (equal); Visualization (equal); Writing‐original draft (equal); Writing‐review & editing (equal). **Andrius Jasilionis:** Data curation (supporting); Investigation (supporting); Methodology (supporting); Validation (supporting); Visualization (equal); Writing‐review & editing (equal). **Olof Böök:** Conceptualization (equal); Funding acquisition (equal); Project administration (equal); Resources (supporting); Supervision (equal); Validation (supporting); Writing‐review & editing (supporting). **Rickard Öste:** Conceptualization (equal); Funding acquisition (lead); Project administration (equal); Resources (equal); Supervision (supporting); Validation (supporting). **Ana Rascón:** Conceptualization (supporting); Project administration (supporting); Supervision (supporting); Writing‐review & editing (equal). **Lovisa Heyman‐Lindén:** Conceptualization (supporting); Project administration (supporting); Supervision (supporting); Writing‐review & editing (supporting). **Olle Holst:** Investigation (supporting); Supervision (supporting); Validation (supporting); Writing‐review & editing (supporting). **Eva Nordberg Karlsson:** Conceptualization (equal); Investigation (equal); Methodology (supporting); Project administration (lead); Resources (equal); Supervision (lead); Validation (equal); Writing‐original draft (equal); Writing‐review & editing (lead).

## Data Availability

The data generated and analyzed during this study are included in this published article.
